# Parent Management Training Combined with Group-CBT Compared to Parent Management Training Only for Oppositional Defiant Disorder Symptoms: 2-Year Follow-Up of a Randomized Controlled Trial

**DOI:** 10.1007/s10578-021-01306-3

**Published:** 2022-01-28

**Authors:** Maria Helander, Pia Enebrink, Clara Hellner, Johan Ahlen

**Affiliations:** 1 Division of Psychology, Department of Clinical Neuroscience, Karolinska Institutet, Nobels väg 9, 171 65 Solna, Sweden; 2 Stockholm City Council Child- and Adolescent Psychiatry, Stockholm, Sweden; 3 Department of Clinical Neuroscience, Centre for Psychiatry Research, Karolinska Institutet & Stockholm Health Care Services, Stockholm County Council, Stockholm, Sweden; 4 Centre for Epidemiology and Community Medicine, Stockholm County Council, Stockholm, Sweden; 5 Department of Global Public Health, Karolinska Institutet, Solna, Sweden

**Keywords:** RCT, Oppositional defiant disorder, Intervention, Moderators, Parent training, Child problem-solving skills training

## Abstract

Parent management training (PMT) is recommended treatment for children with oppositional defiant disorder (ODD) and child-directed cognitive behavior therapy (CBT) is also recommended for school-aged children. The current study examined 2-year follow-up effects of parent management training (PMT) combined with the CBT based group intervention Coping Power Program (CPP) compared to PMT only. Results showed long-term effectiveness of both PMT and PMT combined with CPP in reduced disruptive behavior problems and harsh parenting strategies, and increased emotion regulation- and social communication skills. The earlier reported increase in emotion regulation- and social communication skills in the PMT with CPP condition during treatment remained stable while the PMT condition showed continued improvement during the follow-up period. To conclude, PMT with CPP did generally not provide significant benefits at the 2-year follow-up compared to PMT, apart from an improvement earlier in time regarding emotion regulation- and social communication skills.

*Trial registration number* ISRCTN10834473, date of registration: 23/12/2015.

**Supplementary Information:**

The online version contains supplementary material available at 10.1007/s10578-021-01306-3.

Disruptive behavior disorders such as oppositional defiant disorder (ODD) [1] and conduct disorder (CD) [1] have increasingly been recognized as a major public health concern [2] and are associated with a range of comorbid psychiatric disorders such as mood disorders, anxiety disorders, and substance use disorders [3, 4] and with a large financial and societal burden [5, 6]. Oppositional defiant disorder (ODD) [1] is one of the major causes for contact with child- and adolescent psychiatry and has a lifetime prevalence of 10.2% [3] and children with an ODD diagnosis have an increased risk of developing CD and associated antisocial behaviors [7]. Prompt and sustained treatment effect for disruptive behavior disorders is essential to reduce risk of future antisocial development, individual suffering, and the immense societal costs associated with an antisocial development [6].

Parent Management Training (PMT) is recommended treatment for children up to 12 years of age with disruptive behavior disorders [8], a term summarizing both ODD, CD and Disruptive behavior disorder NOS, and has shown medium effect sizes in numerous meta-analyses on reduced disruptive behavior [9–14], medium effect sizes on improved parental strategies [15–17] and small effect sizes on mental health, including parental stress [15, 17]. The long-term effects of PMT on disruptive behavior have been examined in a few meta-analyses showing sustained effects. One meta-analysis, Van Aar et al. [18], included RCT studies and evaluated long-term effects using within-group effect sizes up to three years after treatment. In the Van Aar meta-analysis, including both intervention trials (clinical level of disruptive behavior problems; *n* = 10), pure prevention trials (including children without clinical levels of disruptive behavior; *n* = 3) and trials in between (including children with differing levels of disruptive behavior; *n* = 27), a sustained effect of PMT was identified, regardless of the initial levels of child disruptive behavior problems. When it comes to long-term effects of PMT on parenting strategies examined in a meta-analysis on clinical and sub-clinical disruptive behavior, no parenting program techniques were associated with stronger long-term effects [10].

Another meta-analysis, Fossum et al. [19], included studies on children with clinical levels of disruptive behavior and evaluated the long-term effectiveness of PMT together with other types of treatment modalities (child CBT, PMT with child directed CBT and family focused treatments). Sustained treatment effects were shown on conduct problems in within group comparisons. A limitation with the Fossum study was the inclusion of non-RCT studies and the inclusion of different treatment modalities alongside PMT in the analysis, making the specific long-term effects of PMT harder to distinguish. In conclusion, very few studies on long-term effects of PMT on disruptive behavior disorder symptoms, with intact randomization exists.

Apart from PMT, clinical guidelines recommend child directed treatment, child-CBT, which teaches children anger management and social- and cognitive problem-solving skills [8]. Lochman and colleagues have stated that emotion regulation, specifically anger control, is a key to the successful decrease of conduct problems [20]. Training in social problem-solving strategies has been found to increase emotion regulatory skills that in turn lead to reduced irritability [21]. The ability to monitor and regulate one’s own negative emotions reduces aggressiveness and in fact, the mere awareness of angry emotions and attempts to generate strategies, seems to suffice to decrease aggressive responses [22]. This is of importance since aggressive behavior is found to be related to peer rejection [23] and peer rejection is, together with problems with social information processing, reciprocally associated with further increased aggressive behavior [24, 25]. One type of problem with social information processing that has been associated with peer rejection is hostile attribution bias, i.e., the tendency to attribute benign or ambiguous social situations and cues of others as more hostile than intended, and with a tendency to generate fewer and more hostile responses to social situations [24]. In a recent meta-analysis, a robust association was found between childhood aggression and hostile attribution showing that the relationship between aggressive behavior and hostile attribution was stronger in emotionally engaging situations [26]. In child CBT treatment, the components that target these difficulties include emotion awareness, emotion regulatory skills, social- and cognitive problem-solving skills training and perspective taking.

The effectiveness of PMT combined with child CBT compared to PMT only on disruptive behavior problems has been examined in a few intervention studies: in a group setting for younger children 4–8 years [27, 28] and in an individual format for children aged 8–12 years [29]. One-year follow-ups of child CBT and PMT compared to PMT effectiveness have shown mixed results. Two of the studies showed significantly reduced behavior problems in the combined treatment compared to PMT only [28, 29] while one of the studies showed no significant differences between the combined versus the single treatment in behavioral outcomes [27]. In addition, PMT combined with child CBT has also been found to increase social skills [28, 29] and to improve parental strategies [27]. Long-term effects of group-based child CBT combined with PMT have not been examined for children aged 8–12 on clinical levels of disruptive behavior disorders.

For clinical utility, there is a need to differentiate between treatments that generally work for a disruptive child and her/his family, and how treatments work when individual characteristics are considered. In meta-analyses, PMT programs have been found to be equally effective for families with high and low socioeconomic status immediately post treatment [30, 31], but treatment gains were harder to sustain for disadvantaged families [31]. Parental level of education has further not been found to moderate treatment results [32]. Concerning child characteristics, severity of child behavior problems before PMT is initiated has been associated with larger reductions in behavior problems [32, 33]. A high number of ODD symptoms in childhood has been associated with increasingly poor functioning in relationships with peers, partners and parents in adult life [34]. Child age [35] and gender differences [18] have been reported not to moderate treatment effects in meta-analyses examining PMT effectiveness in clinical and subclinical populations. Further, a common comorbid diagnosis with disruptive behaviors is Attention Deficit Hyperactivity Disorder (ADHD) [1, 4]. Comorbid ADHD has not been found to moderate treatment effects of PMT in an individual participant data meta-analysis [36]. To conclude, a firm knowledge base supports short term treatment effectiveness of PMT for treatment of children with disruptive behavior. There is however a need for studies that shed light on the long-term effectiveness of PMT in clinical samples, as well as PMT in combination with group child CBT, and studies that investigate the moderating effect of baseline characteristics of children.

The current study presents data from a 1- and 2-year follow-up of a clinical trial conducted in Swedish child- and adolescent psychiatry [37, 38]. In the trial, children aged 8–12 diagnosed with ODD, CD or disruptive behavior NOS, were randomized to (1) the Swedish group-based PMT program KOMET or (2) to KOMET combined with the child group-based CBT program, Coping Power Program (CPP), in this article called KOMET with CPP. Komet is a Swedish group-based PTP program for parents that consists of 11 group sessions of two and a half hours each with 6 families (parents of 6 target children) in each group [39]. The Komet program includes the treatment components found in most PMT programs aiming to increase positive parent–child interaction and reduce disruptive behavior: Play or positive time together with the child, training in giving clear instructions/commands, praise and rewards to increase reinforcement on positive behavior, reducing the reinforcement of negative behavior by not focusing on minor disruptive behaviors, handling anger outburst calmly, and using non-punitive consequences. The child CBT program used was the child-component of CPP [40]. The CPP is a manual-based group CBT intervention for children 8–14 years old. In CPP, children are trained in emotion regulation, anger management skills, social problem-solving skills, perspective taking, social skills and handling group pressure.

As previously reported, the effectiveness pre- to post treatment showed no significant differences in behavior problems post treatment, while social skills were significantly increased in the KOMET with CPP group compared to KOMET only [38]. In moderator analyses, children with high levels of behavior problems, manifested by high number of ODD diagnostic criteria fulfilled according to clinician-rated diagnostic interview, and children with high levels of clinician-rated risk for future antisocial development, benefitted significantly more from the combined treatment group compared to PMT only in reduced behavior problems. In addition, the group of children with a high number of clinician-rated ODD symptoms benefitted more from the combined treatment compared to PMT only in significantly increased social skills.

## Aims and Research Hypothesis

The overall aim of the present study was to investigate the treatment effects from treatment termination to the 2-year follow-up of KOMET with CPP compared to KOMET only in reducing child conduct problems and increasing social skills, as well as in improving parenting behaviors. Our first hypothesis was that the combined treatment, KOMET with CPP would be more effective in reducing child disruptive behavior compared to KOMET only in the long-term. Our second hypothesis was that the significantly increased social skills at post assessment for children in the KOMET with CPP-group compared to KOMET, would be sustained during follow-up. Our third hypothesis was that the combined treatment would yield less parental stress and improved parental strategies compared to PMT only during the follow-up period. Our fourth hypothesis was that for children with severe ODD, the significantly reduced behavior problems and increased social skills post treatment would remain during the follow up period. We further hypothesized that there would be no moderator effect of comorbid ADHD, prescribed medication at baseline, or gender.

## Method

### Trial Design

The study was a 2-year follow-up of a clinical study with a randomized controlled design. In the present study, the data analyzed are parent rated questionnaires collected at baseline (T1), post treatment (T2), one year after treatment termination (T3), and two years after treatment termination (T4). The study was approved by the regional ethics review board in Stockholm and registered on Current Controlled Trials www.isrctn.com (ISRCTN10834473).

### Participants

**Fig. 1:**
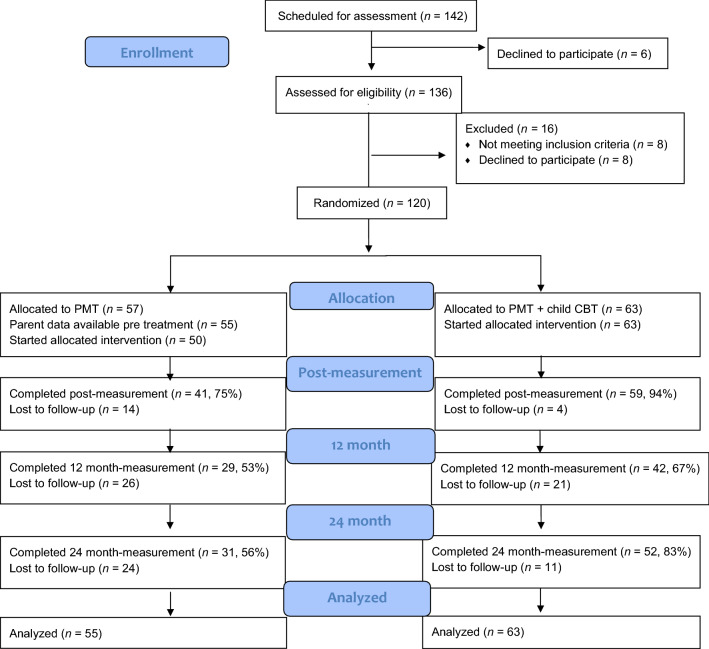
Flow chart

A total of 120 children, 8–12 years old, diagnosed with ODD, ODD combined with CD or disruptive behavioral disorder NOS, were enrolled in the study. Exclusion criteria were (a) autism (b) intellectual disability or (c) severe other psychiatric comorbid disorders that required treatment. See Table 1 for participant characteristics. For the present study, parent-rated baseline data were available for 118 children (KOMET, n = 55 and KOMET with CPP, n = 63), since in two cases, parent-rated data was lost at baseline due to an administrative mistake. In the KOMET condition 29 (52.7%) of the families randomized participated in assessment at T3 and 31 (56.4%) at T4. In the KOMET with CPP condition, 42 (66.7%) of the families randomized participated in assessment at T3 and 52 (82.5%) at T4. In Fig. 1, the flow of participants from inclusion to the 2-year follow-up is illustrated.

**Table 1:** Characteristics and demographics of participants included in the study

Characteristics	Total *n* = 118 M (SD)/*n* (%)	KOMET *n* = 55 M (SD)/*n* (%)	KOMET with CPP *n* = 63 M (SD)/*n* (%)	Statistics	
				t- test/χ^2^ KOMET vs. KOMET with CPP	p-value
Child characteristics					
Mean age (SD)	9.34 (1.22)	9.36 (1.29)	9.33 (1.16)	t(114) = .11	.91
Number of boys	88 (74.6%)	40 (72.7%)	48 (76.2%)	χ^2^ (1*)* = *.*19	.66
Mean ODD symptoms (SD)	5.38 (1.41)	5.29 (1.44)	5.46 (1.40)	t (116) = − .65	.52
Children with ODD diagnosis	106 (89.8%)	48 (87.3%)	58 (92.1%)	χ^2^* (1)* = .74	.39
Children with 7 or 8 ODD symptoms	27 (22.9%)	12 (21.8%)	15 (23.8%)	χ^2^* (1)* = .07	.80
Children with ODD and comorbid CD	5 (4.2%)	2 (3.6%)	3 (4.8%)	χ^2^ *(1)* = *.*09	1.00*
Children with DBD NOS diagnosis	12 (10.2%)	7 (12.7%)	5 (7.9%)	χ^2^* (1)* = .74	.39
Children with ADHD	78 (66.1%)	35 (63.6%)	43 (68.3%)	χ^2^* (1)* = .28	.60
Parent/family characteristics					
Parents with post graduate education	56 (47.5%)	27 (49.1%)	29 (46.0%)	χ^2^* (1)* = .11	.74

ODD = Oppositional Defiant Disorder; DBD NOS = Disruptive behavior disorder not otherwise specified (in this study children who fulfill 3 diagnostic criteria of ODD); 7 or 8 ODD symptoms = children who fulfill 7 or 8 DSM-5 diagnostic criteria for ODD; CD = Conduct disorder; ADHD = Attention Deficit Hyperactivity Disorder; Parents with post graduate education = Proportion of parents with university level of education

*p-value based on Fisher’s Exact Test

### Procedure

Children were recruited from six child- and adolescent psychiatric outpatient clinics in mid-Sweden. For the present study of follow-up effects, parents filled out rating scales through an internet-based secure homepage. The follow-up assessments were part of the trial design, to which the parents had given their consent. The parents were given the option to fill out rating scales in paper format, and if that format was preferred the material was sent via regular mail with a pre-payed return envelope. In the case parents did not fill out the questionnaires, contact was made via phone to see if help was needed with login, other technical support or if there were other reasons for not completing the questionnaires. In the case both parents had filled out the rating scales at all time points, data from one parent was randomly chosen to be included in the dataset. In the case one parent had filled out the rating scales at more time-points than the other, this parent was chosen to be part of the follow-up dataset. Compared to the pre-post study, 18 of the parents were interchanged to include the parent that participated with most data.

### Treatments

The PMT program used in this study was the Swedish PMT program KOMET [39]. KOMET is a group treatment for parents, led by two group leaders, consisting of 11 group sessions of two and an half hours each with six families (parents of 6 children) in each group. The child CBT program used was a Swedish adaption of the child-component of the Coping Power Program, a manual-based group CBT intervention for children 8–12 years old [40, 41].

### Measures

#### Primary Outcome Measure

The primary outcome in the present study was the oppositional/defiant subscale of the Disruptive Behavior Disorder rating scale rated by parents (DBD-ODD) [42]. The ODD subscale consists of eight items that corresponds to the diagnostic criteria of the ODD diagnosis ranging from range from 0–3, from “not at all “ to “very much”. (Chronbach’s alphas at T1-T4 ranged from *α* = 0.79 to 0.90 from pre to 2 years follow-up).

#### Secondary Outcome Measures

Social skills were measured with three different measures. The first, the Social Competence Scale-Parent version (P-COMP) [43], was a 12-item-scale that assesses child emotion regulation skills such as “controlling temper when there is a disagreement” and prosocial communication skills such as “resolves problems with friends alone”. The measure has a 5-point scale, from “not at all” to “very well” (*α* = from 0.84 to 0.90). The second measure was the SDQ prosocial scale [44] which captures a child’s capacity to consider other people’s feelings and child helpfulness towards others such as “helpful if someone is being hurt” and “kind to younger children” (*α* = from 0.70 to 0.75). The third measure was a modified version of the Social Skills Rating System (SSRS) [45], which captures prosocial competence such as helping others, complying with rules, asking others for information, ability to communicate with adults and responding appropriately to teasing. In the present study the total scale was used (*α* = from 0.82 to 0.90).

To assess parental strategies, we used the Parenting Practices Interview (PPI) [46]. In the present study, two subscales were used: the Harsh and inconsistent disciplines subscale that assesses harsh as well as submissive responses to child misbehavior (15 items, *α* ranged from 0.76 to 0.83) and the Praise and Incentives subscale (11 items, *α* ranged from 0.70 to 0.74) that evaluates the extent to which parents responds with hugs, praises, and rewards when a child shows desired or expected behavior.

To assess parental stress, we used the Perceived Stress Scale (PSS) [47]. This is a 14-item scale targeting the degree to which situations in life are appraised as stressful. In the present study a short version of 10 items was used (range 0–4 from “never” to “very often”, *α* ranged from 0.84 to 0.89).

#### Moderators/Predictors of Treatment Effects

The moderators investigated in this study were clinician-rated severity of ODD, ADHD, prescribed medicine at baseline, and gender. The severity of ODD at baseline was evaluated with the *Kiddie-SADS, Present and Lifetime Diagnosis (Version P/L),* a semi-structured diagnostic interview [48]. The assessments were made by psychologists with several years of experience from clinical child and adolescent psychiatry. To identify children with the most severe problem level, the number of ODD diagnostic criteria fulfilled was used to divide the sample into two groups: (1) light to moderate problems with three to six diagnostic criteria fulfilled (*n* = 91) and (2) severe problems with seven to eight diagnostic criteria fulfilled (*n* = 27). This dichotomous variable was then entered as a moderator in the regression analyses. In addition to the dichotomization into two groups, we also explored number of criteria as a continuous moderating variable (e.g., values between three and eight). ADHD diagnosis at baseline was evaluated with the *Kiddie-SADS.* Prescribed medicine was measured using a dichotomous question (yes/no) at baseline, where parents were asked if their child had any prescribed medicine that they took regularly.

### Data Analysis

Statistical analyses were performed in SPSS version 25 and in the R software program [49]. Linear mixed models (LMM) were applied to analyze the long-term effects of the two treatments. LMMs are adequate when analyzing repeated measurement data and also involve the benefit of not deleting participants with an incomplete number of observations [50]. In the analysis of long-term treatment effects, we performed the LMMs in two ways. First, we examined treatment effects over the whole time period (T1 to T4). Due to the partly large amount of attrition during the follow-up period, we additionally explored attrition and its possible effect on the long-term outcome. Following the procedure described by Hedeker and Gibbons [50], we entered a dummy-coded variable of attrition (that is, missing or non-missing at the 2-year follow-up) into the LMM evaluating the outcome. Then, we specifically examined the treatment effects over the follow-up period (T2 to T4) in segmented LMMs, that is, partitioning the time variable into a separate treatment-interval, and a follow-up interval. Additional analyses controlling for the attrition effects were not possible to perform on the follow-up period as participants with missing data did not contribute to the follow-up data. Effect sizes (Cohen's *d*) were estimated based on the beta-estimates obtained from the LMMs. Thus, within-group effect sizes were calculated by multiplying the beta-coefficient of time with number of months (to estimate the change in scores over the whole time period), and dividing with the pooled standard deviation of the measure at the pre-assessment [51]. Between-group effect sizes were calculated similarly, however, by using the time by treatment interaction beta-coefficient. To explore possible effects of the heterogeneity of the study sample, we additionally ran sensitivity analyses for all outcome measures excluding participants with a comorbid conduct disorder and participants with a DBD-NOS diagnosis.

Further, we examined the Reliable Change Index (RCI) [52] to interpret the clinical significance of the effect in parent-rated ODD symptoms. Children whose parents completed the 2-year follow-up assessment were categorized according to four categories: recovered; improved, unchanged, or deteriorated. Children who were reliably changed between T1 and T4 and moved from a clinical population to a non-clinical population were categorized as recovered. A reliable change was defined as the difference in the DBD-ODD scale between T1 and T4, divided by the standard error of the measure. An RCI below -1.96 or above 1.96 was considered a reliable statistical change at *p* < 0.05. A clinical population was defined as scores at or above the 95th percentile on the ODD-scale [42], gender and age-specific, in a Swedish normative sample (unpublished data). Children who showed a reliable decrease but did not move from a clinical to a non-clinical population were categorized as improved. Children with no reliable change were categorized as unchanged, and children with a reliable increase of symptoms were categorized as deteriorated. Differences between groups were not tested using inferential statistic tests due to low power. We described the proportions of children in the different categories for both treatment arms separately, and also by subdividing into high or low clinician-rated baseline ODD.

### Attrition Effects

Of the 118 children with complete baseline data, 71 (60.2%) participated in the one-year follow-up and 83 (70.3%) participated in the 2-year follow-up. A Chi-square test showed no significant difference between the two treatment conditions in proportion of participants lost to follow-up at one-year follow-up (KOMET *n* = 26 [47.3%], and KOMET with CPP *n* = 21 [33.3%]). However, at the 2-year follow-up there was a significantly larger proportion lost to follow-up in the KOMET condition (*n* = 24, [43.6%] compared to KOMET with CPP *n* = 11, [17.5%], *p* = 0.002). To understand differences in patterns of attrition between the two treatment conditions, we analyzed baseline descriptive statistics by missingness separately for each treatment arm (see Table 2). First, in the KOMET condition only, a Chi-square test showed that a larger proportion of girls than expected was missing at the 2-year follow-up. No such difference was found in the KOMET with CPP condition. Further, in the group missing in the KOMET condition, there were significantly higher levels of baseline disruptive behaviors (DBD-ODD), significantly lower emotion regulation- and prosocial communication skills (P-COMP), and significantly higher skills in the positive parental strategies using praise and incentives (PPI Praise) compared to completers at the 2-year follow-up assessment. In the KOMET with CPP condition, there was significantly lower baseline prosocial competence (SSRS) in the group missing at follow-up, compared to completers. This indicates that families in the KOMET condition who completed the follow-up assessment differed from the original sample, with lower levels of child behavior problems, higher child emotion regulation- and social communication skills as well as more reported use of parental praise and incentives.

**Table 2:** Attrition analysis of baseline data for study completers versus families missing at the 2-year follow-up

Baseline variables	KOMET			KOMET + CPP		
	Completers^a^ (*n* = 31)	Missing^b^ (*n* = 24)	*p*-value ^c^	Completers^a^ (*n* = 52)	Missing^b^ (*n* = 11)	*p*-value ^c^
Girls (%)	16.1%	41.7%	.035*	23.1%	27.3%	.714
Higher Education (%)	58.1%	37.5%	.130	51.9%	18.2%	.052
ODD risk (%)	19.4%	25.0%	.615	21.2%	36.4%	.435
DBD-ODD (Mean (SD))	12.5 (3.5)	15.8 (5.0)	.008**	13.6 (4.0)	15.5 (3.4)	.113
SDQ prosocial (Mean (SD))	6.0 (2.0)	6.1 (1.9)	.815	5.8 (2.1)	5.1 (1.0)	.100
P-COMP (Mean (SD))	30.6 (7.1)	25.3 (6.7)	.007**	28.0 (5.7)	25.1 (6.7)	.195
SSRS (Mean (SD))	49.2 (10.5)	45.0 (8.4)	.106	47.6 (10.7)	39.9 (7.5)	.011*
PSS (Mean (SD))	26.3 (5.2)	28.7 (5.7)	.118	28.4 (6.7)	30.6 (6.6)	.321
PPI Harsh (Mean (SD))	36.0 (9.4)	35.1 (11.5)	.752	30.8 (9.4)	38.5 (13.7)	.100
PPI Praise (Mean (SD))	34.2 (8.8)	39.0 (7.3)	.033*	35.2 (9.9)	37.5 (7.1)	.364

^a^Families with data at the 24-month follow-up assessment

^b^ Families with missing data at the 24-month follow-up assessment

^c^Difference between Completers and Missing. Welch two sample t-tests for continuous variables and Fisher’s Exact Test for categorical variables

*p < .05, **p < .01, Higher Education % = Proportion university level of education compared to elementary + high school level of education. ODD-risk % = Percentage children with severe ODD; DBD ODD = The Parent/Teacher Disruptive Behavior Disorder rating scale – Oppositional Defiant Disorder scale; P-COMP = Social Competence Scale- Parent; SSRS = Social Skills Rating System total scale; SDQ prosocial = Strengths and Difficulties Questionnaires Prosocial scale; PSS = Perceived Parental Stress; PPI Harsh = Parenting Practices Interview, Harsh and inconsistent discipline scale; PPI Praise = Parenting Practices Interview, Praise and incentives scale

## Results

### Long-Term Effects on Behavior Problems

To examine our first hypothesis, if the combined treatment KOMET with CPP was more effective in reducing disruptive behaviors compared to KOMET over the 1- and 2-year follow-up, we evaluated parent-rated ODD-symptoms in the DBD-ODD scale in a series of LMMs. In the LMMs, the KOMET condition was entered as the reference category. Means and standard deviations of the included outcomes in raw score format are presented in Table 3.

**Table 3:** Means and standard deviations in PMT and PMT with child CBT from pre-treatment to two years follow up, and between-group effect sizes (standardized mean differences, Cohen’s d) at the 2-year follow-up

Measure	PMT				PMT with Child CBT				2 YEAR Between group effect size
	PRE n = 55	POST n = 41	1 YEAR n = 29	2 YEAR n = 31	PRE n = 63	POST n = 59	1 YEAR n = 42	2 YEAR n = 52	
	M (SD)	M (SD)	M (SD)	M (SD)	M (SD)	M (SD)	M (SD)	M (SD)	Cohen’s d^a^ [95% CI]
Child behavior problems									
DBD ODD	13.95 (4.49)	11.30 (5.00)	11.07 (5.34)	9.03 (4.15)	13.94 (3.98)	10.86 (4.73)	10.54 (5.49)	10.90 (5.79)	-0.37 [-0.82, 0.08]
Child social skills									
P-COMP	28.29 (7.34)	30.50 (7.82)	32.96 (7.44)	33.43 (7.05)	27.52 (5.92)	33.14 (8.10)	32.24 (8.34)	33.22 (7.96)	-0.02 [-0.47, 0.42]
SDQ Prosocial	6.05 (1.93)	6.29 (2.21)	5.90 (2.54)	6.39 (2.32)	5.70 (2.01)	6.63 (2.0)	5.90 (2.04)	5.73 (2.15)	-0.30 [-0.74, 0.15]
SSRS	47.40 (9.76)	48.63 (11.10)	50.61 (9.90)	52.66 (15.49)	46.20 (10.56)	51.39 (12.08)	49.10 (13.54)	49.94 (12.76)	-0.19 [-0.64, 0.25]
Parental skills and competences									
PPI Harsh	35.60 (10.24)	24.90 (7.70)	26.54 (7.31)	28.29 (9.84)	32.10 (10.53)	23.69 (8.21)	26.57 (10.17)	25.40 (9.03)	0.31 [-0.14, 0.75]
PPI Praise	36.27 (8.44)	41.90 (7.72)	37.61 (7.73)	37.29 (8.76)	35.59 (9.46)	39.81 (8.61)	37.71 (7.52)	36.96 (7.68)	-0.04 [-0.48, 0.40]
PSS	27.28 (5.47)	24.66 (7.05)	23.93 (7.04)	25.70 (6.27)	28.77 (6.67)	25.10 (6.58)	25.98 (6.65)	25.62 (6.69)	0.01 [-0.43, 0.46]

^a^A negative effect-size indicate an advantage to the PMT condition

DBD ODD = The Parent/Teacher Disruptive Behavior Disorder rating scale – Oppositional Defiant Disorder scale, P-COMP = Social Competence Scale-Parent, SSRS = Social Skills Rating System total scale, SDQ prosocial = Strengths and Difficulties Questionnaires Prosocial scale; PPI harsh = Parenting Practices Interview harsh and inconsistent discipline; PPI Praise = Parenting Practices Interview praise and incentives scale; PSS = Perceived Parental Stress

In the primary outcome (DBD-ODD), the LMM showed a significant main effect of time over the whole time period, pre to 24 months follow up (*t*_274.6_ = − 4.51, *p* < 0.001, *d* = − 0.80), interpreted as a large reduction in ODD-symptoms (i.e., in the reference treatment condition). We found no significant time by treatment interaction effect, meaning no significant difference between treatments was found from pretreatment over the follow-up period. When entering the dichotomized variable of attrition in the LMM, we found similar results (i.e., significant main effect of time and no significant time by treatment interaction effect when controlling for attrition).

When examining the follow-up period from post-assessment to the 2-year follow-up, we found a significant main effect of time in the KOMET condition (*t*_257.6_ = − 2.07, *p* = 0.040, *d* = − 0.38), interpreted as a small reduction in ODD symptoms during the follow-up period. Further, we found a significant time by treatment interaction (*t*_255.7_ = 2.08, *p* = 0.039, *d* = 0.49), between post and 2 years follow-up. When rerunning the same analysis using the KOMET with CPP as the reference category, we found no significant main effect of time from post to follow-up in the KOMET + CPP condition. Thus, the results showed that the KOMET with CPP condition remained stable from post to follow-up while the KOMET condition improved significantly during the follow-up period reaching a similar result as the combined treatment.

### Long-Term Effects on Social Skills

According to the second hypothesis, we examined whether the significantly increased social skills seen during treatment in KOMET with CPP compared to KOMET in the original study would remain at the 2-year follow-up. Regarding child emotion regulation- and social communication skills as measured by P-COMP we found a significant main effect of time over the whole time period (*t*_273.2_ = 2.78, *p* = 0.006, *d* = 0.50) but no significant time by treatment effect, indicating that both treatment conditions showed moderately increased emotion regulation and prosocial communication skills between T1 and T4. Similar results were found when running the subsequent LMM controlling for attrition. Looking at the follow-period only, we found no significant main effect of time, nor time by treatment interaction effect.

When it comes to child capacity to consider other people’s feelings and child helpfulness as measured by SDQ prosocial scale, we observed no significant main effect of time, nor time by treatment interaction effect. Consequently, no long-term effect on the SDQ prosocial scores was found between T1 and T4. Similar results were found when controlling for attrition. Further, we found no significant main effect of time over the 2-year follow-up period, however, a significant time by treatment interaction effect (*t*_262.8_ = − 2.32, *p* = 0.020, *d* = − 0.51). When rerunning the same analysis using the KOMET with CPP as the reference category, we found a significant main effect of time from post to follow-up in the KOMET + CPP condition (*t*_262.8_ = − 3.57, *p* < 0.001, *d* = − 0.59). Thus, the results showed that the significant increase in SDQ prosocial during the treatment period in the KOMET + CPP condition regressed during the follow-up period while the KOMET condition remained unchanged.

Finally, regarding prosocial competence as measured by SSRS, we found a significant main effect of time over the whole time period (*t*_273.2_ = 2.22, *p* = 0.028, *d* = 0.39) but no time by treatment interaction effect, indicating that both conditions showed a small increase in prosocial competence between T1 and T4. However, when controlling for attrition, the main effect of time was no longer significant (*t*_261.9_ = 1.70, *p* = 0.090, *d* = 0.31). Over the follow-up period we found no significant main effect of time, however, a significant time by treatment interaction effect (*t*_256.2_ = − 2.02, *p* = 0.045, *d* = − 0.45), indicating that the KOMET + CPP condition showed a relative decrease in prosocial competence compared to the KOMET condition during the follow-up period.

### Long-Term Effects on Parenting Strategies and Stress

According to the third hypothesis, we examined if the KOMET with CPP would yield improved parental strategies and less parental stress compared to KOMET at the 2-year follow-up. Regarding harsh parenting, we found a significant main effect of time over the whole time period (*t*_273.8_ = − 3.46, *p* < 0.001, *d* = − 0.53) but no significant time by treatment interaction effect, indicating a moderate decrease in harsh parenting over the whole time period in both treatment conditions. Similar results were found when controlling for attrition. No significant main effect of time, or time by treatment interaction effect was found over the follow-up period, meaning no significant changes in harsh parenting in any of the treatment conditions over the follow-up period.

When examining parental praise, we found no significant main effect of time, nor time by treatment interaction effect over the whole time period, meaning none of the treatment conditions showed changes in parental praise between T1 and T4. Similar results were found when controlling for attrition. We found a significant small main effect of time over the follow-up period (*t*_263.7_ = − 2.82, *p* = 0.005, *d* = − 0.42), indicating a small decrease in parental praise. No time by treatment interaction effect was found, indicating no significant difference between treatment conditions over the follow-up period. When it comes to parental stress, we found no significant main- or interaction effect, neither over the whole time period nor over the follow-up period.

### Moderators of Long-Term Effects

Regarding the moderator high vs. low ODD severity, 22.9% of the children were rated high in ODD severity at baseline (21.8% [*n* = 12] in the KOMET condition and 23.8% [*n* = 15] in the KOMET with CPP condition). At the 2-year follow-up, 20.5% of the families had children with a high number of ODD criteria at baseline, 19% [*n* = 6] in the KOMET condition, and 21% [*n* = 11] in the KOMET with CPP condition.

When entering the dichotomous variable high/low to moderate baseline ODD in the LMM evaluating DBD-ODD (over the whole period), we found a significant three-way interaction effect of time × treatment × high/low baseline ODD (*t*_268.9_ = − 2.46, *p* = 0.014). This indicated that the difference between treatments over time was moderated by high ODD-symptoms. Regarding the different measures of social skills (P-COMP, SSRS, SDQ-prosocial), we found a significant three-way interaction effect of time × treatment × high/low ODD on the P-COMP (*t*_266.4_ = 1.98, *p* = 0.049) and the SDQ-prosocial subscale (*t*_275.7_ = 2.95, *p* = 0.003). However, when exploring severity of ODD at baseline as a continuous variable, we found no significant moderation effect (three-way time × treatment × ODD criteria interaction for DBD-ODD, P-COMP, SDQ-prosocial, or SSRS.

A total of 66.1% of the children had ADHD at baseline (63.6% [*n* = 35] in the KOMET condition and 68.3% [*n* = 43] in the KOMET with CPP condition). When entering ADHD in the LMMs evaluating DBD-ODD, P-COMP, SSRS, and SDQ-prosocial over the whole period, we found no evidence that ADHD moderated the effect for any of the outcomes.

In 43.2% of the children, parents reported them having a prescribed medication at baseline (36.4% [*n* = 20] in the KOMET condition and 49.2% [*n* = 31] in the KOMET with CPP condition). We found no evidence that prescribed medication at baseline moderated the effect on DBD-ODD; P-COMP, SSRS, or SDQ-prosocial over the whole period.

Regarding gender, 27.3% were girls in the KOMET condition and 23.8% in the combined condition at baseline, whereas 16.1% (*n* = 5) of the girls that remained in the study were in the KOMET condition and 23.1% (*n* = 12) in the combined condition at the 2-year follow-up. We entered the dichotomous variable gender as moderator in the LMMs evaluating DBD-ODD and the three measures of social skills. We found a significant moderating effect only in one outcome, the SSRS where girls in the KOMET with CPP conditions benefitted significantly more compared to girls in the KOMET condition (*t*_276.8_ = 2.28, *p* = 0.020).

### Sensitivity Analyses

When excluding the participants who met criteria for CD and participants with a DBD-NOS diagnosis at baseline, we found no important differences in the results compared to the results of the full sample (Supplementary Tables S1 and S2). The only observed differences were that, although similar effect-sizes, the main and interaction effects on DBD-ODD, and the interaction effect on SSRS did not reach statistical significance.

### Clinically Significant Change

To additionally explore our first hypothesis regarding change in disruptive behavior, we analyzed clinically significant change for the primary outcome, DBD-ODD. In the sub-sample of children with complete data (*N* = 83), it appeared that children in the KOMET with CPP condition recovered to a larger degree whereas there was no difference between the conditions for those who improved (see Table 4). A larger proportion in the KOMET condition was unchanged compared to the KOMET with CPP group. Further, a larger proportion had deteriorated in the KOMET with CPP condition compared to the KOMET condition. Furthermore, in an exploratory subgroup analysis of clinically significant change divided by level of clinician-rated ODD symptoms at baseline, it appeared that children with high number of ODD symptoms at pre-assessment improved more in the KOMET with CPP condition, while children with low to moderate ODD were similarly recovered/improved in both treatment condition, but fewer deteriorated in the KOMET condition, see Table 4.

**Table 4:** Clinically significant according to Jacobson and Truax (1991) between baseline and 24-months follow up in participants with complete data at two years follow-up

Sample	Category	KOMET *n* (%)	KOMET + CPP *n* (%)
Total sample (*n* = 83)	Recovered	8 (26%)	18 (35%)
	Improved	7 (23%)	10 (19%)
	Unchanged	14 (45%)	15 (29%)
	Deteriorated	2 (6%)	9 (17%)
Low to moderate ODD at baseline (*n* = 66)	Recovered	8 (32%)	14 (34%)
	Improved	5 (20%)	7 (17%)
	Unchanged	11 (44%)	11 (27%)
	Deteriorated	1 (4%)	9 (22%)
Severe ODD at baseline (*n* = 17)	Recovered	0 (0%)	4 (36%)
	Improved	2 (33%)	3 (27%)
	Unchanged	3 (50%)	4 (36%)
	Deteriorated	1 (17%)	0 (0%)

Measure DBD ODD = The Parent/Teacher Disruptive Behavior Disorder rating scale – Oppositional Defiant Disorder scale

Recovered = Children who were reliably changed between T1 and T4, and moved from a clinical population to a non-clinical population; Improved = children who showed a reliable decrease, but did not move from a clinical to a non-clinical population; Unchanged = children with no reliable change; Deteriorated = children with a reliable increase in symptoms; Low to moderate ODD at baseline = children who fulfill 3 to 6 DSM-5 diagnostic criteria for ODD; Severe ODD at baseline = children who fulfill 7 or more DSM-5 diagnostic criteria for ODD

## Discussion

This study aimed to evaluate the long-term treatment effects of KOMET combined with CPP compared to KOMET only in children, 8–12 years, with disruptive behavior disorders in a clinical child- and adolescent psychiatric context. In this article, the follow-up effects from post-treatment to 2-year follow-up are described. At baseline, there were no significant differences between the groups in any of the outcome variables. At post-treatment, as we have previously reported, a significant time x group interaction benefitting KOMET with CPP compared to KOMET was found in two measures of social skills, PCOMP and SDQ prosocial. This implies that the two groups differed in the social skills outcomes at post-treatment with a larger improvement seen in KOMET with CPP.

Our first hypothesis was that children in KOMET with CPP would benefit more in terms of reduced disruptive behavior over time compared to KOMET. This hypothesis was not confirmed. Over time, there were no significant differences between the two treatment conditions in parent-rated ODD symptoms, the DBD-ODD scale from T1 to T4. When comparing the results of the behavioral outcome in this study to previous studies examining the additive effect of child CBT to PMT for the same age group, this study did not show as clear results as those shown by Kazdin et al. [29], where parent-rated disruptive behavior in the individual PMT with child CBT condition improved more compared to the individual PMT condition at the 1-year follow-up. Our results are more similar to the Norwegian study by Larsson et al. [27] where adding group child CBT to PMT showed no additional effect compared to PMT only for younger children.

In an exploratory calculation of proportion with reliable clinical change in DBD-ODD in the subsample with complete data, a larger proportion of children in the KOMET with CPP condition had recovered or improved compared to the children in the KOMET only condition. However, the proportion of children that had deteriorated was also larger in the KOMET with CPP group compared to KOMET. The proportion of deteriorated children in PMT with CPP is in line with rates of deterioration found in other studies on treatment effects of PMT at 18 [53] and 24 months follow-up [54]. Some of the variations in reliable clinical change could be explained by the level of difficulties in disruptive behavior before treatment, where children with high levels of clinician-rated ODD symptoms were recovered and improved to a larger extent in the KOMET with CPP condition. The large proportion of children missing at the 2-year follow-up in the KOMET condition in the high ODD group, (50%), compared to the KOMET with CPP condition (27%), restricts our possibilities to draw firm conclusions.

The second hypothesis was that children in KOMET combined with CPP would show increased social skills compared to children in KOMET only and that this effect would remain over time. This hypothesis was not confirmed since there were no significant differences between the two conditions in any measure of social skills, and the only measure of social skills that improved significantly from baseline to 2-year follow-up was the measure of emotion regulation- and social communication skills (PCOMP). The PCOMP results are in proximity to those of Drugli et al. [55] and Webster Stratton and Hammond [28], where both PMT and PMT with child CBT showed significant improvements in social skills over time but there were no difference between treatment conditions at the one-year follow-up.

When it comes to time-point of improvement in emotion regulation and social communication, a difference between the treatments conditions was detected where the children in the KOMET with CPP improved during the treatment period while children in the KOMET condition reached the same treatment gains but over a longer period of time. It is not surprising to see an early and sustained effect in emotion regulation and social communication in PMT with CBT since emotion regulation skills explicitly were trained in the Coping Power Program, and sustained effects have been demonstrated in earlier research on child CBT combined with PMT as outlined above. More surprising was the effect, however delayed, in the KOMET condition only. The results might be influenced by the fact that those who were missing at two years follow-up in the KOMET condition had significantly lower PCOMP ratings at baseline compared to those who remained in the study. It might however also be the case that PMT by itself results in improved child emotion regulation- and social communication skills. This is supported by a study by Hagen and Ogden [56] showing that low pre-treatment social skills predicted larger improvement in social skills following PMT post-treatment.

Contrary to expected, the significant improvement in KOMET with CPP during the treatment period in child helpfulness and capacity to consider others’ feelings as measured by the SDQ prosocial scale regressed during the follow-up period. SDQ prosocial targets more of an emotionally empathic capacity that is not explicitly trained in Coping Power Program, compared to the extensive training in emotion regulation techniques and this might explain the regression. No effect at all was seen in the KOMET group which is not consistent with an earlier PMT study where an effect on SDQ prosocial behavior was seen over time [57]. Likewise surprising, no treatment effect was found in SSRS over time after controlling for attrition. SSRS is a multidimensional measure of social skills whereas SDQ prosocial and PCOMP each measure different single dimensions of social skills [58]. Using the total score of SSRS as in this study, might conceal effects in the different dimensions of social skills. The result might however imply that PMT as well as PMT with child CBT produce immediate treatment effects but do not result in long-lasting effects two years after treatment in measures of child helpfulness and capacity to consider others’ feelings and prosocial behavior. Long-term evaluations of PMT effects on social skills are scarce and there is, to our knowledge, no other study with intact randomization of PMT effects in a clinical sample with data on child social skills two years post treatment.

The third hypothesis, that the combined treatment would yield less parental stress and further improve parental strategies compared to PMT only, was not confirmed. At post-treatment, the groups in both treatment arms had improved with regard to reduced harsh parenting, increased parental praise and reduced parental stress. At two years follow-up, the initial reduction of harsh parenting in both treatment groups was stable but parental praise went back to pre-treatment levels, and parental stress showed a small but non-significant decrease over time. This result is not seen in the literature where previous studies comparing child CBT with PMT compared to PMT only on parental skills and parental stress have shown significant effects over the treatment period that have been sustained during one-year follow-ups in both treatment groups [27, 28]. In addition, the Kazdin study showed that PMT with child CBT was significantly better than PMT only in the parental outcomes [29]. The difference in results may however be explained by the longer follow-up period in current study. Furthermore, positive parenting has been suggested a key factor, mediating change in child behavior problem in a study by Gardner et al. [59], however on a short-term basis. Studies of long-term PMT effects are scarce and when long-term key parenting components were examined in a meta-analysis, no parenting program techniques were associated with stronger long-term effects [10]. More studies with long-term follow-up of PMT effects on disruptive behavior with regarding type of parental strategies are needed.

The fourth hypothesis, that children with high levels of ODD would benefit more from the KOMET with CPP condition compared to the KOMET condition at the 2-year follow-up was confirmed. However, this result must be considered with caution since it is derived from a smaller group of children with severe ODD at baseline and there was a large proportion of these children missing in the KOMET condition at the 2-year follow-up. Moreover, when number of baseline ODD diagnostic criteria fulfilled was calculated as a continuous moderator, no moderator effect was found, adding further uncertainty to the result. It is not obvious that the numbers of diagnostic criteria can be treated as a continuous variable but, as this result indicates, there was no linear relationship between number of ODD symptoms and treatment effectiveness while there might be a cumulative effect where children with a high number of ODD symptoms might have a larger effect. Further studies on this subgroup with a large number of ODD symptoms are needed to draw firm conclusions.

We found no evidence that children with ADHD, or children that regularly took medication, benefitted more (or less) from the treatments compared to children without ADHD and medication. Also, we found no evidence of differential effects between the two treatments dependent on these two variables (i.e., ADHD and medication). These results are in line with a recent review concluding that there is no evidence that ADHD would reduce the effect of behavioral interventions on conduct problems [60]. Also, a recent treatment study on children with CD found that medication for ADHD did not moderate treatment efficacy [61].

Gender was found to moderate social competencies as measured by SSRS showing a larger treatment effect for girls in the KOMET with CPP condition compared to KOMET. This result needs to be taken with caution since there was a significant dropout of girls in the KOMET condition at the 2-year follow-up.

Looking at treatment effects over time, it seems as if the different treatment conditions were equally effective in reducing disruptive behavior and improving emotion regulation- and social communication skills, as well as in reducing harsh parenting. The only difference in effects between the two treatment arms that was detected was an earlier improvement in time in emotion regulation and social communication in KOMET with CPP compared to KOMET. An earlier improvement in emotion regulation and social communication might still be an important factor since it may help disruptive children to handle conflicts more efficiently and become more accepted in social contexts with prosocial peers and thereby reduce the risk of peer rejection. Training in emotion awareness, emotion regulation, and social- and cognitive problem-solving skills are likely to affect the irritability symptoms in ODD such as tantrums more directly, compared to PMT where tantrums are handled with reduced attention as well as with more indirect methods such as improved relationship with parents, attention to prosocial behavior and to give clear and prepared instructions.

The findings in this study are important in the attempt to shed more light on the effects of adding child CBT to PMT. This study is the first to investigate the 2-year follow-up effect of group CBT combined with PMT compared to PMT only for children 8–12 years with disruptive behavior disorders two years after treatment. The effectiveness of PMT combined with child CBT compared to PMT has been investigated previously at one-year post-treatment showing mixed results as shown above. One way of understanding the difference in effects could be the different treatment formats, group vs. individual delivery. However, the question of format has been explored in a study where individual delivery of the Coping Power program was compared to group delivery on parent-rated disruptive behavior, finding no differences between the treatment modalities at one and four years post treatment on parent-rated disruptive behavior while teacher-ratings showed that children with lower initial self-regulation improved more in the individual format [62, 63]. Thus, the number of studies examining the additive effect of child CBT to PMT is still small, and there are differences in format, age group addressed, as well as treatment results that still obscures a firm conclusion.

### Limitations

There are several limitations to this study. One obvious being the large amount of attrition in the KOMET group at the 2-year follow-up, leaving 56% of the participants in the KOMET group and 83% in the KOMET with CPP group, which poses a threat to internal validity [64]. This missingness leaves us with some uncertainty regarding the treatment effects. It is not possible to rule out that the large group of children missing at the 2-year follow-up in the KOMET group with significantly larger pretreatment difficulties in the primary outcome, would have impacted the long-term treatment effect in the KOMET group if they were to remain in the study. Another limitation was that the study was underpowered at start with 120 participants of the 130 anticipated, and power was further reduced by the large attrition. Moreover, the results at the 2-year follow-up are likely confounded by the natural maturation of the children and life events not controlled for in this study. Further, when exploring prescribed medication as a moderator, we only had dichotomous data on which children that regularly took medication and no specification on what medication. Finally, a limitation is the lack of follow-up data from the children themselves. It would have been valuable to gain information about the usability of the skills trained in the Coping Power Program over time.

## Conclusions

The 2-year follow-up of KOMET compared to KOMET with CPP for children with disruptive behavior disorder, 8–12 years old, showed that the Swedish parent management training program KOMET was effective in reducing disruptive behavior, increasing emotion regulation and prosocial communication, and reducing harsh parenting strategies and that these effects hold over a 2-year follow-up period. In the group where the child CBT program Coping Power Program was added to KOMET, an improvement was seen in emotion regulation and prosocial communication skills in the KOMET with CPP group during the treatment period while children in the KOMET condition improved and reached the same result during the follow-up period. The study has also shed some light on the long-term effects of PMT on parental strategies and stress which is a valuable contribution in the field of PMT research where long-term follow-up studies are scarce. The study points out a need for future studies on the effects of child CBT combined with PMT, long-term effects of differing parental strategies in PMT outcomes, how the social skills trained in child CBT are sustained over time and if PMT by itself produces a sufficient increase in social skills.

## Summary

For children with oppositional defiant disorder (ODD) up to twelve years of age, Parent management training (PMT) is recommended treatment and for school-aged children, child-directed cognitive behavior therapy (CBT) is also recommended. The current study examined the 2-year follow-up effects of PMT combined with the child CBT intervention Coping Power Program (CPP) compared to PMT only in a randomized controlled trial in Swedish Child- and Adolescent Psychiatric setting. Participants were one hundred and eighteen children, 8–12 years, with ODD, CD, or Disruptive Behavioral Disorder NOS. The results showed long-term effectiveness of both PMT and PMT combined with CPP in reduced disruptive behavior problems and harsh parenting strategies, and increased emotion regulation- and social communication skills. In the group where the child CBT was added to PMT, an improvement was seen in emotion regulation and prosocial communication skills during the treatment period while children in the PMT condition improved and reached the same result during the follow-up period. To conclude, PMT with CPP did not in general provide significant benefits at the 2-year follow-up compared to PMT, apart from an improvement earlier in time regarding emotion regulation- and social communication skills.

## Supplementary Information

Below is the link to the electronic supplementary material.

Supplementary file1 (DOCX 21 KB)
