# Supplementary material for: Parent Management Training Combined with Group-CBT Compared to Parent Management Training Only for Oppositional Defiant Disorder Symptoms: 2-Year Follow-Up of a Randomized Controlled Trial
Source: Child Psychiatry Hum Dev. 2022 Jan 28;54(4):1112–26. doi: 10.1007/s10578-021-01306-3 (PMC10271908; doi:10.1007/s10578-021-01306-3)
Supplement: Supplementary file 1 — Supplementary file1 (DOCX 21 KB) [file 10578_2021_1306_MOESM1_ESM.docx]

**Table S1**

*Original Linear Mixed models compared to a sensitivity analysis in a restricted sample (i.e., excluding participants with CD and DBD-NOS) over the whole time period, pre – 24 months follow up.*

| Outcome | Effect^a^ | Whole sample (*N*=118) | | | Sensitivity sample (*N*=101) | | |
| --- | --- | --- | --- | --- | --- | --- | --- |
|  |  | t-value | p-value | Cohen’s d | t-value | p-value | Cohen’s d |
| DBD-ODD | Main | **-4.51** | **< .01** | **-0.80** | **-4.40** | **< .01** | **-0.90** |
|  | Interaction | 1.52 | .13 | 0.34 | 1.82 | .07 | 0.46 |
| P-COMP | Main | **2.78** | **.01** | **0.50** | **2.58** | **.01** | **0.53** |
|  | Interaction | -0.26 | .79 | -0.06 | -0.25 | .80 | -0.07 |
| SDQ prosocial | Main | 0.39 | .69 | 0.06 | 0.26 | .80 | 0.05 |
|  | Interaction | -1.21 | .23 | -0.25 | -1.28 | .20 | -0.29 |
| SSRS | Main | **2.22** | **.03** | **0.39** | **2.00** | **.05** | **0.41** |
|  | Interaction | -1.07 | .28 | -0.24 | -1.09 | .28 | -0.28 |
| PSS | Main | -0.51 | .61 | -0.09 | -0.75 | .46 | -0.14 |
|  | Interaction | -0.91 | .36 | -0.20 | -0.77 | .44 | -0.18 |
| PPI Harsh | Main | **-3.45** | **< .01** | **-0.57** | **-3.71** | **< .01** | **-0.63** |
|  | Interaction | 1.02 | .31 | 0.20 | 1.83 | .07 | 0.39 |
| PPI Praise | Main | 0.18 | .86 | .03 | 0.01 | .99 | 0.00 |
|  | Interaction | -0.04 | .97 | -.01 | 0.28 | .78 | 0.06 |

Notes: ^a^ Main effect of time (i.e., the effect of the KOMET only treatment) and interaction effect of treatment*time (i.e., the added effect for the KOMET + CPP). DBD ODD = The Parent/Teacher Disruptive Disorder Behavior rating scale – Oppositional Defiant Disorder scale, P-COMP = Social Competence Scale-Parent, SSRS = Social Skills Rating System total scale, SDQ prosocial = Strengths and Difficulties Questionnaires Prosocial scale; PPI Harsh = Parenting Practices Interview harsh and inconsistent discipline; PPI Praise = Parenting Practices Interview praise and incentives scale; PSS = Perceived Parental Stress

Table S2

*Original Linear Mixed models compared to a sensitivity analysis in a restricted sample (i.e., excluding participants with CD and DBD-NOS) over the follow-up time period, post – 24 months follow up.*

| Outcome | Effect | Whole sample (*N*=118) | | | ODD-Sample (*N*=101) | | |
| --- | --- | --- | --- | --- | --- | --- | --- |
|  |  | t-value | p-value | Cohen’s d | t-value | p-value | Cohen’s d |
| DBD-ODD | Main | **-2.07** | **.04** | **-0.38** | -1.74 | .08 | -0.37 |
|  | Interaction | **2.08** | **.04** | **0.49** | 1.74 | .08 | 0.46 |
| P-COMP | Main | 1.25 | .21 | 0.23 | 1.08 | .28 | 0.24 |
|  | Interaction | -1.18 | .24 | -0.28 | -1.12 | .26 | -0.30 |
| SDQ prosocial | Main | 0.12 | .91 | 0.02 | 0.00 | 1.00 | 0.00 |
|  | Interaction | **-2.32** | **.02** | **-0.51** | **-2.20** | **.03** | **-0.53** |
| SSRS | Main | 1.56 | .12 | 0.30 | 1.29 | .20 | 0.29 |
|  | Interaction | **-2.02** | **.04** | **-0.49** | -1.72 | .09 | -0.48 |
| PSS | Main | 1.28 | .20 | 0.23 | 0.44 | .66 | 0.09 |
|  | Interaction | -0.49 | .363 | -0.11 | -0.30 | .76 | -0.08 |
| PPI Harsh | Main | 1.87 | .06 | 0.27 | 0.88 | .38 | 0.14 |
|  | Interaction | -0.15 | .88 | -0.03 | 0.59 | .56 | 0.12 |
| PPI Praise | Main | **2.82** | **.01** | **-0.42** | **2.75** | **>.01** | **-0.48** |
|  | Interaction | 0.52 | .60 | 0.10 | 1.05 | .30 | 0.23 |

Notes: ^a^ Main effect of time (i.e., the effect of the KOMET only treatment) and interaction effect of treatment*time (i.e., the added effect for the KOMET + CPP). DBD ODD = The Parent/Teacher Disruptive Disorder Behavior rating scale – Oppositional Defiant Disorder scale, P-COMP = Social Competence Scale-Parent, SSRS = Social Skills Rating System total scale, SDQ prosocial = Strengths and Difficulties Questionnaires Prosocial scale; PPI Harsh = Parenting Practices Interview harsh and inconsistent discipline; PPI Praise = Parenting Practices Interview praise and incentives scale; PSS = Perceived Parental Stress
